# Proof of concept in utilizing *in-trans* surface display system of *Lactobacillus plantarum* as mucosal tuberculosis vaccine via oral administration in mice

**DOI:** 10.1186/s12896-018-0461-y

**Published:** 2018-10-11

**Authors:** Anhar Danial Mustafa, Jeevanathan Kalyanasundram, Sarah Sabidi, Adelene Ai-Lian Song, Maha Abdullah, Raha Abdul Rahim, Khatijah Yusoff

**Affiliations:** 1 Department of Cell and Molecular Biology, Faculty of Biotechnology and Biomolecular Sciences Universiti Putra Malaysia, 43400 Serdang, Selangor Darul Ehsan Malaysia; 2 Department of Microbiology, Faculty of Biotechnology and Biomolecular Sciences, Universiti Putra Malaysia, 43400 Serdang, Selangor Darul Ehsan Malaysia; 3 Department of Pathology, Faculty of Medicine and Health Science, Universiti Putra Malaysia, 43400 Serdang, Selangor Darul Ehsan Malaysia; 4 Institute of Bioscience, Universiti Putra Malaysia, 43400 Serdang, Selangor Darul Ehsan Malaysia; 5 Malaysia Genome Institute, 43000 Kajang, Selangor Darul Ehsan Malaysia

**Keywords:** *M. tuberculosis* vaccine, Surface display vaccine, *L. plantarum*, *In-trans approach*

## Abstract

**Background:**

Tuberculosis is one of the most common and deadliest infectious diseases worldwide affecting almost a third of the world’s population. Although this disease is being prevented and controlled by the Bacille Calmette Guérin (BCG) vaccine, the protective efficacy is highly variable and substandard (0–80%) in adults. Therefore, novel and effective tuberculosis vaccine that can overcome the limitations from BCG vaccine need to be developed.

**Results:**

A novel approach of utilizing an *in-trans* protein surface display system of *Lactobacillus plantarum* carrying and displaying combination of *Mycobacterium tuberculosis* subunit epitope antigens (Ag85B, CFP-10, ESAT-6, Rv0475 and Rv2031c) fused with LysM anchor motif designated as ACERL was constructed, cloned and expressed in *Esherichia coli* Rossetta expression host. Subsequently the binding capability of ACERL to the cell wall of *L. plantarum* was examined via the immunofluorescence microscopy and whole cell ELISA where successful attachment and consistent stability of cell wall binding up to 4 days was determined. The immunization of the developed vaccine of *L. plantarum* surface displaying ACERL (Lp ACERL) via the oral route was studied in mice for its immunogenicity effects. Lp ACERL immunization was able to invoke significant immune responses that favor the Th1 type cytokine response of IFN-γ, IL-12 and IL-2 as indicated by the outcome from the cytokine profiling of spleen, lung, gastrointestinal tract (GIT), and the re-stimulation of the splenocytes from the immunized mice. Co-administration of an adjuvant consisting of *Lactococcus lactis* secreting mouse IL-12 (LcIL-12) with Lp ACERL was also investigated. It was shown that the addition of LcIL-12 was able to further generate significant Th1 type cytokines immune responses, similar or better than that of Lp ACERL alone which can be observed from the cytokine profiling of the immunized mice’s spleen, lung and GIT.

**Conclusions:**

This study represents a proof of concept in the development of *L. plantarum* as a carrier for a non-genetically modified organism (GMO) tuberculosis vaccine, which may be the strategy in the future for tuberculosis vaccine development.

**Electronic supplementary material:**

The online version of this article (10.1186/s12896-018-0461-y) contains supplementary material, which is available to authorized users.

## Background

Tuberculosis has latently affected almost a third of the world’s population with *Mycobacterium tuberculosis*, the causative agent for tuberculosis. It was estimated that in 2016, out of the 10.4 million infected cases, 1.3 million tuberculosis related-deaths were reported worldwide [1]; over 95% of which occur in low- and middle-income countries, predominantly in Africa, South East Asia and Eastern Europe [2]. The disease is being prevented and controlled by the Bacille Calmette Guérin (BCG) vaccine, the only viable vaccine approved for human use since 1921. Nevertheless, the protective efficacy of the BCG vaccine has been highly variable and sub-standard (0–80%) in adults especially in endemic tropical and sub-tropical regions, due to pre-existing immune responses to the vaccine [3]. Therefore, there is a strong need for a new and effective tuberculosis vaccine to be developed either as a replacement or as a booster in order to overcome the limitations from the current BCG vaccine.

At present, research for novel tuberculosis vaccination strategies are intensely focused on the attenuated mycobacterial vaccines as well as subunit and viral vector vaccines [4]. Among these approaches, the subunit vaccine approach has shown to give the most promising outcome as indicated by its success in stage I or II clinical trials [5]. This approach is regarded to have a higher safety approval but typically suffers from the lack of any significant protective immune response and requires adjuvant in order to improve dramatically the immunogenicity response [6]. Meanwhile, a different approach towards developing an effective tuberculosis vaccine is to use the probiotic lactic acid bacteria (LAB) as a mucosal delivery vehicle, which exhibits the antigenic protein(s) on their cell surface. This strategy has already been successfully performed with promising results in giving protective immunity against rotavirus, group B streptococcus, human papillomavirus virus 16-induced tumors, tetanus, chicken anemia virus and enterovirus [7]. The growing interest in the use of the LAB protein surface display system for mucosal vaccination purposes is due to strong requirement for effective strategies in delivering vaccine antigens, microbiocides and therapeutics to the mucosal tissues [7] in which the *M. tuberculosis* infection target entry site is at the mucosal lining of the respiratory tract. By focusing on this route for vaccine administration, effective protection gain by the mucosal cells against the pathogen can be achieved primarily by the enhancement of mucosal cells to vaccine interaction whilst having reduced potential side effects when compared to systemic routes of administration [8]. This is because studies have shown that protection against mycobacterial infection were conferred by mucosal immunity that induces both mycobacterial-specific T helper cells-1 (Th-1) and secretory IgA responses which are the key immune response against *M. tuberculosis* [9, 10]. Moreover, this approach provides painless and easy vaccine administration with higher compliance rate than the other known invasive administration methods.

In this study, candidate mucosal tuberculosis vaccine of ACERL utilizing *in-trans* protein surface display system of *Lactobacillus plantarum* was constructed, cloned and expressed in *Esherichia coli* Rossetta expression host. Subsequently, this expressed fusion protein was harvested and purified before being externally attached onto the cell wall of *L. plantarum*. This strategy of using an independent expression host for protein expression and external attachment of the expressed protein onto the cell wall of the intended bacterial carrier is referred as the *in-trans* surface display system concept. The recombinant ACERL protein was studied for its functionality in its binding capability to the cell wall of *L. plantarum* and its immunogenicity effects based on mouse animal model. The effect of adding adjuvant consisting of *Lactococcus lactis* secreting IL-12 co-administered with the ACERL fusion antigen was also determined as it has the potential to induce a more favorable condition than that of ACERL only for further generating significant and improved protective immune responses. Thus, this study is intended to determine the potential response using LAB of *L. plantarum* as a non-GMO vaccine carrier via the *in-trans* surface display system for the development of candidate mucosal tuberculosis vaccine.

## Results and discussion

### Construction of ACERL fusion antigen for display onto *L. plantarum* cell surface

**Fig. 1:**
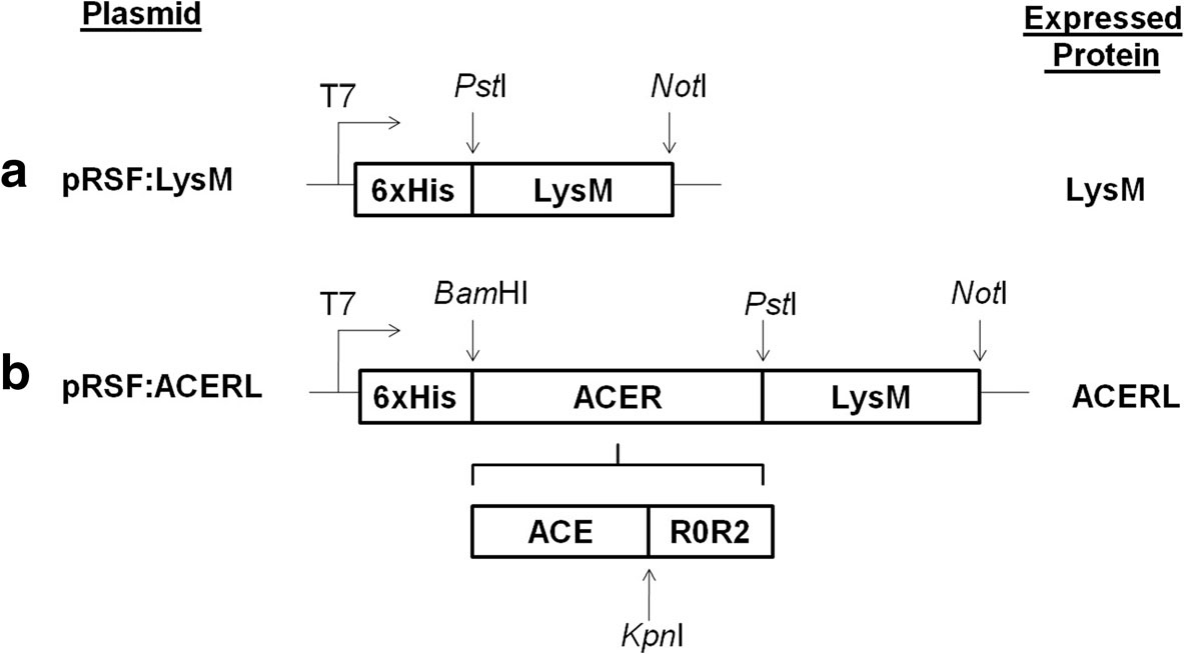
Cloning strategy using pRSF:LysM (**a**) as plasmid backbone for constructing the desired expression plasmid of pRSF:ACERL (**b**). Plasmid pRSF:LysM was constructed initially before the fusion and inclusion of antigen of interest ACER gene was performed

LysM anchor motif was used for the surface display of the ACER fusion antigen, and a schematic outline of the generated expression vector is provided in Fig. 1. The expression plasmid was constructed initially with the construction of pRSF:LysM as the plasmid backbone before the insertion of the ACER gene that formed the desired ACERL fusion gene. The fusion enables ACER protein to non-covalently attach to the cell wall of *L. plantarum* via the N-terminal cell wall anchor of LysM.

The display of proteins on LAB using LysM anchor domain for vaccine development is well known and has been extensively reviewed [7, 11]. For example, Moeini *a*nd colleagues have utilized LysM anchor domain to develop an oral chicken anemia virus vaccine based on live non-recombinant *L. acidophilus* [12] and Varma and co-workers also used the same motif to develop an oral candidate vaccine against hand-foot-and-mouth disease [11]. These studies showed promising results in achieving significant levels of antigen-specific serum antibodies in the animal models. However, the novelty of the present study lies in the application of the *in-trans* surface display concept where the non-covalently anchor protein of LysM domain was fused to the recombinant *M. tuberculosis* antigen of ACER to enable extracellular attachment to live *L. plantarum*. This strategy ensures that a non-GMO exhibiting a *M. tuberculosis* antigen on its cell surface [13] was achieved. To date, only Kuczkowska *a*nd colleagues have investigated the potential of using LAB surface displaying the tuberculosis antigen for mucosal immunization [14]. However, their approach did not utilize the *in-trans* surface display concept as presented in this study. Instead, they have engineered the *L. plantarum* to surface display the tuberculosis antigen via intracellular attachment to the bacterial cell wall. Although the results indicate significant induction of antigen-specific proliferative responses in lymphocytes purified from tuberculosis-positive donors, this strategy still involved a genetically engineered *L. plantarum* carrier, which is a GMO and may not be acceptable to some.

In this study, different types of *M. tuberculosis* antigens of Ag85B, CFP-10, ESAT-6, Rv2031 and Rv0475 were chosen since these antigens are considered immunodominant proteins with ability to induce protective immune responses in several different animal models [15, 16] as well as in human beings [17]. The combinations of these immunodominant antigens as multiple antigen fusion protein, similar to the approach of the current tuberculosis subunit trial vaccines [18], can represent a broad epitopic repertoire that leads to improved activation of helper as well as cytotoxic T cell responses. These combinations have been shown to significantly improve the immunogenicity and protective efficacy of subunit vaccines [19]. Moreover, previous study by Piubelli *a*nd co-workers indicated that a multiple immunogenic antigen of a subunit vaccine was more likely to be immunoreactive than a single immunogenic protein [20], hence better immune responses can be accomplished.

### Expression of recombinant clones

**Fig. 2:**
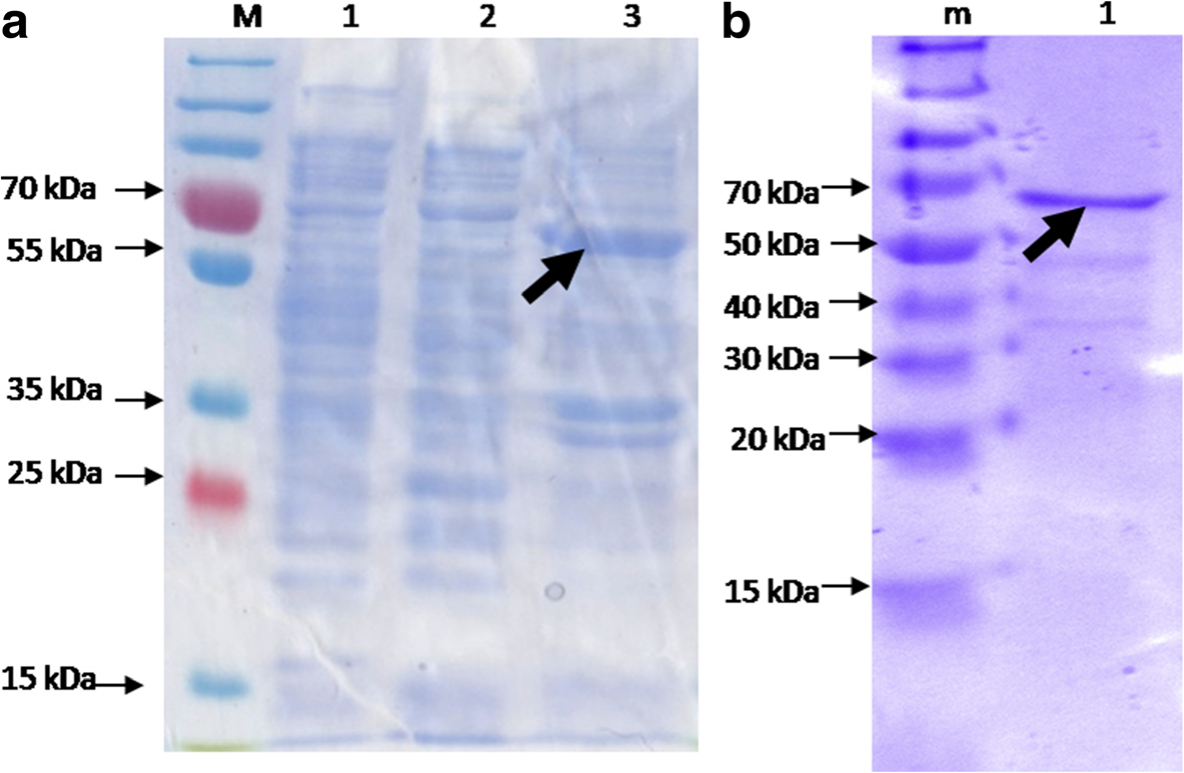
Detection of ACERL produced from *E. coli.* **a** Over-expression of recombinant fusion proteins of ACERL expressed from *E. coli* Rossetta (DE3) host via SDS-PAGE analysis. Cells were induced with 1 mM/ml IPTG, agitated for 6 h at 30 °C.Label 1–3 indicates the protein of interest ACERL expression profiles whereLane M: PageRuler™ PrestainedPlus Protein Ladder (Fermentas, Canada); Lane 1: Uninduced ACERL; Lanes 2: Induced ACERL from the intracellular fraction; Lanes 3: Induced ACERL from the lysate pellet fraction. ACERL was observed in the lysate pellet fraction only as indicated by the arrow. **b** SDS-PAGE of soluble fraction of ACERL treated with 5% NLS. Lane m: PageRuler™ Unstained Protein Ladder (Fermentas, Canada); Lane 1: ACERL intracellular fraction; Soluble recombinant ACERL proteins was achieved and detected at ~ 64 kDa respectively as indicated by arrow

The recombinant *E.coli* Rossetta (DE3) pLysS cells harboring the constructed plasmid pRSF:ACERL was induced with 1 mM/mL IPTG and verified by SDS-PAGE approach. SDS-PAGE analysis showed that the ACERL fusion protein was over-expressed in *E. coli* as indicated in Fig. 2a where a prominent protein band corresponding to the predicted molecular masses of approximately 64 kDa was observed. As expected, the negative control which was *E. coli* Rossetta (DE3) pLysS harboring pRSFDuet-1 plasmid did not show any over-expressed protein band. Unfortunately, the expressed ACERL fusion protein was observed to be localized in the inclusion bodies (IBs) of the host. This can be due to the sub-optimal expression parameters used or the characteristics of the protein themselves that have a high tendency to form IBs when expressed in a foreign host [21]. Thus, a simpler and faster way of solubilizing the IBs in *E. coli* was performed instead, which utilizes the N-laurlyl sarcosine treatment. The NLS treatment approach in solubilizing ACERL localized in the IBs of the host system was achieved by the use of 1% NLS for 24 h at 20 °C (see Fig. 2b). This treatment was able to solublize the IBs without any denaturation and renaturation process. The supernatants containing the solubilized recombinant proteins of interest were collected and purified on Ni^2+^ affinity columns of His-spin trap column (Merck, USA). The NLS treated recombinant proteins of ACERL was purified and concentrated from 0.1 g cell wet mass with protein recovered of 0.84 ± 0.02 mg (Table 1) and protein recovery and extractability of 38.2%. However, the purity was not 100% as there were few faint protein bands shown in SDS-PAGE gel of Fig. 2b.

**Table 1:** Recombinant antigen protein recovery of ACERL from inclusion bodies (IBs) by 5% (*w*/*v*) N-lauroyl sarcosine (NLS) and its purification by Ni^2+^-NTA affinity chromatography

Stages of solubilization /purification	Protein (mg)
Recombinant antigen	ACERL
Cell lysate (0.1 g cell wet mass)	3.74 ± 0.08
Insoluble fraction	2.20 ± 0.1
Soluble fraction (after 5% NLS treatment)	1.31 ± 0.1
Purified (after Ni2 + -NTA purification)	0.84 ± 0.02
Extractability (%)	38.2%

The overly expressed ACERL was shown to be deposited and accumulated into the inclusion bodies (IBs) of the *E. coli* host (Fig. 2) which are dense and porous particles containing almost exclusively over-expressed and aggregated proteins [21]. This aggregation of proteins can be due to the nature of the over-expressed and non-native proteins being partially folded, misfolded or a combination of both [22]. Most the *M. tuberculosis* antigens which are currently being explored for the development of the tuberculosis subunit vaccine are commonly purified from such IBs groups [20, 23, 24]. This is most probably due to the nature of the *M. tuberculosis* antigen which has a higher inclination for incorrect protein folding and aggregation when over-expressed in a heterologous expression system such as *E. coli* [20]. To overcome this issue, the use of NLS, an ionic and non-denaturant detergent, has been shown to effectively solubilize IBs composed of various protein types [22, 25, 26]. The NLS penetrates the IBs through pores, disrupting the aggregated proteins from binding to their hydrophobic patches, thereby preventing interaction and aggregation between these proteins [26]. Overall recovery of the purified recombinant antigens of interest using this single-step purification method was approximately > 98% pure as shown in SDS-PAGE analysis (Fig. 2b). Meanwhile the protein recovery yield was 38.2%, for ACERL (see Table 1). Extractability of ACERL via the NLS treatment was shown to be better than the typical denaturation and renaturation processed which commonly yielded 15–25% protein recovery from IBs [21].

### Anchoring and stability of recombinant *M. tuberculosis*-LysM protein onto *L. plantarum* cell wall

**Fig. 3:**
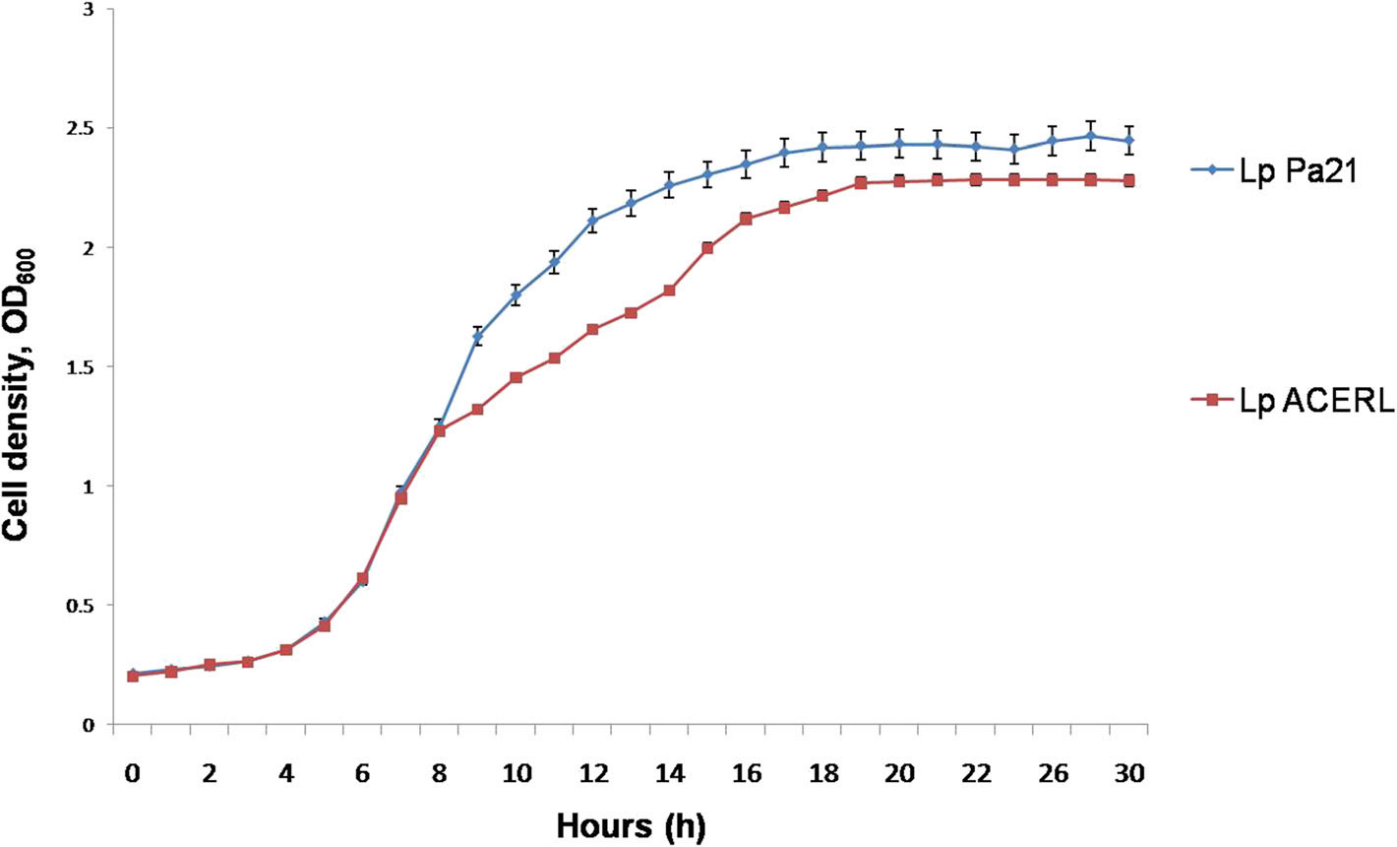
Growth profiles of *L. plantarum* Pa21 carrying ACERL antigen in MRS broth without antibiotic. Interaction for binding on *L. plantarum* was performed at OD_600_ 0.5. The doubling time of Lp was the highest compared to *L. plantarum* carrying antigens ACERL or referred as Lp ACERL. Meanwhile Lp Pa21 was the wild-type host control. Each sample was analyzed in triplicates

The growth study of *L. plantarum* interacting with the recombinant antigen ACERL, was performed in a period of 30 h as shown in Fig. 3. This growth study on post-interaction with the recombinant antigen was performed in order to determine the effect of cell wall binding by recombinant antigen ACERL on the *L. plantarum* bacterial development and survivability. Based on the growth profile, the wild-type *L. plantarum* Pa21 (Lp) has the shorter doubling time of 34.4 min while the *L. plantarum* with surface displayed antigen of ACERL was shown to have higher doubling time of 39.5 min. Based on the increase in doubling time of *L. plantarum* carrier cell, the surface display attachments of the recombinant proteins did influence the physiological aspect of the carrier cell. It was postulated that larger protein size of ACERL when attached onto the cell surface of *L. plantarum* may induce significant stresses to the *L. plantarum* cell that leads to a reduction in cell division and growth. This result was in accordance with a study by Fredriksen and co-workers that showed surface displayed *Yersinia pseudotuberculosis* invasin antigens onto the cell wall of *L. plantarum* via several types of anchor motif including LysM exhibited significant reduction in the growth rate towards the bacterial carrier from strains harboring plasmids with Lp_1452- or Lp_1568-derived anchors [27]. They discovered that both the over-expression and the types of recombinant antigens used may provide significant burden and stress on the host physiological state that leads to the detrimental effect on the host growth rate. Nonetheless, no significant adverse effects were found on the growth and survivability of *L. plantarum* surface displaying ACERL as indicated by the steady increased of cell density until 18 h of incubation followed by a plateau of growth trend (stationary phase) from the period of 18 to 30 h of incubation.

**Fig. 4:**
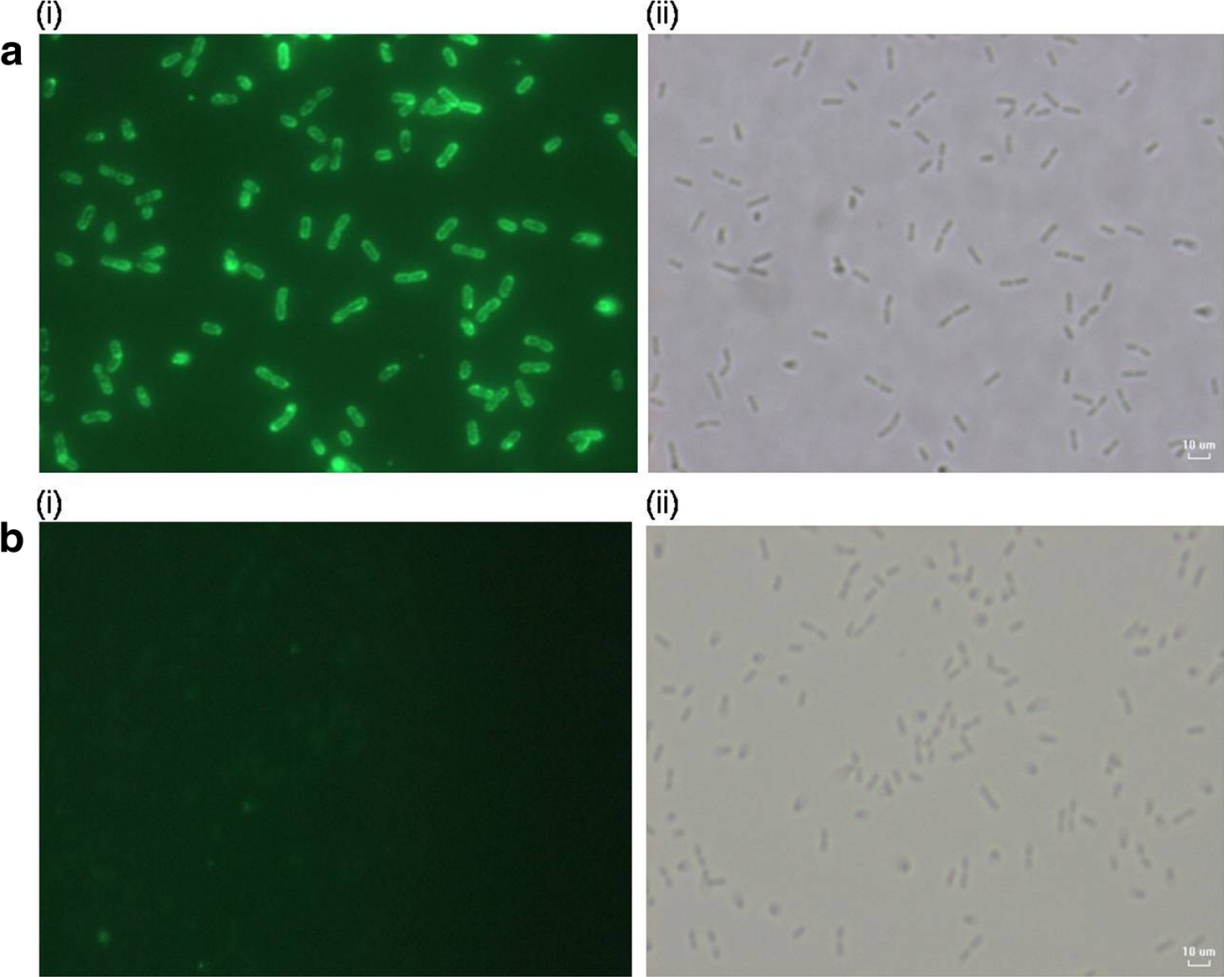
Immunofluorescence microscopy of *L. plantarum* interacted with ACERL protein (**a**) and 1× PBS as negative control (**b**). (i) FITC conjugated secondary antibody staining for *L. plantarum* interacted with ACERL protein or 1× PBS while (ii) is phase contrast outcome for *L. plantarum* interacted with ACERL protein (**a**) or 1× PBS (**b**). The positive FITC signalling by *L. plantarum* cells interacted with ACERL protein indicated successful docking of the target protein to live *L. plantarum* cell wall. Note that *L. plantarum* displayed ACERL was probed with mouse anti-his primary antibody and stained with anti-mouse IgY secondary antibody conjugated with FITC. (Images were observed under: × 100 oil immersion objective)

Immunofluorescence staining was used to validate the binding capability of the fusion proteins onto the cell wall of *L. plantarum*. This analysis enabled the visualization for confirming the protein binding structure of LysM fused with ACER was functional and correctly expressed. By using mouse anti-his monoclonal as primary antibody and secondary antibody of goat anti-mice conjugated FITC, the bacterial cells incubated with the fusion protein of ACERL was shown to exhibit consistent green fluorescence signal as shown in Fig. 4a. The green fluorescence signals which were overlapped with the corresponding lactobacilli cells in the phase contrast image indicated the presence of the recombinant proteins of interest on the *L. plantarum* cell, thus validating the binding capability of the recombinant protein of interest that were shown to anchor onto the cell wall surface of *L. plantarum*. Meanwhile, the control bacterial cells showed no observable green fluorescence signal (Fig. 4b).

**Fig. 5:**
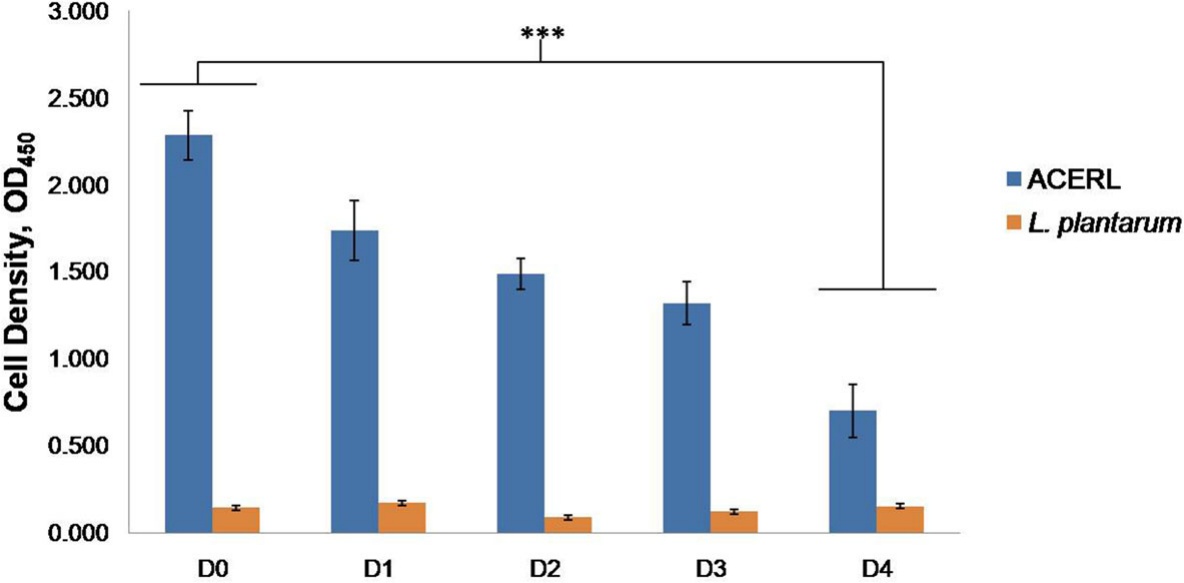
Analysis of the binding of the fusion proteins onto the cell surface *L. plantarum* via whole cell ELISA for a four-day period. D0: initial values; D1 – D4: the days of assay. *L. plantarum* attached with ACERL was subjected to ELISA using mouse monoclonal anti-His as primary and HRP conjugated anti-mouse as secondary antibody. Negative control was set to be *L. plantarum* incubated with 1× PBS. Each groups were analyzed in triplicates. Results are presented as mean ± SD of the mean (Abs) with mean values of *p* < 0.001 = *** between the groups of Day 4 with the groups of Day 0 are significantly different

Investigation on the stability of ACERL fusion protein to be able to anchor and display on the *L. plantarum* cell surface was performed via whole cell ELISA for over a 4-day period as shown in Fig. 5. The absorbance values (OD_450_ nm) correlated, relatively, to the frequency of the fusion proteins that had attached onto the cell wall of *L. plantarum*, thus reflecting the protein binding stability each day from Day 0 to 4. The *L. plantarum* with attached ACERL showed consistent binding stability patterns with gradual decrease of absorbance values until Day 4, a significant reduction of binding frequency by about 40% from the initial binding of the attached fusion protein until Day 4 (*P* < 0.001) as observed in Fig. 5. Based on the results in Fig. 4 and 5, both methods corroborated with the findings that the attachment of the recombinant protein of interest to the *L. plantarum* cell wall was successfully achieved without detrimentally affecting the growth of the *L. plantarum* as its bacterial carrier (Fig. 3).

Interestingly, the frequency of ACERL that was attached to the *L. plantarum* cell wall based on the immunofluorescence and whole cell ELISA analysis showed similar pattern to previous studies [12, 28] where consistent binding within a period of 2 days was observed. However, as shown in Fig. 4, gradual decrease in the absorbance values over a period of 4 days indicated that the binding stability of the attached recombinant antigen maybe reduced with longer periods of incubation time. This reduction in the binding may be due to proteolytic effect of Lactobacilli housekeeping proteases such as HtrA [29] which cleaves the cell wall attached with the recombinant antigen of interest. Moreover, the occurrence of cell division may also cause the detachment of bound recombinant antigen of interest to *L. plantarum* cell wall; thereby contributing to the decrease in recombinant antigen of interest binding stability.

### Immunogenicity effects of Lp ACERL and Lp ACERL/IL-12 on mouse animal model

The potential use of the LAB for live vaccine delivery of surface displayed recombinant tuberculosis antigen of ACERL with or without IL-12 adjuvant was evaluated via the oral route administration using a mouse animal model. The oral delivery is advantageous considering its superior patient compliance and easy administration with the ability to induce both mucosal and systemic immunities [30, 31]. In addition, *L. plantarum* is also a natural resident of the intestinal microflora thus; it would be the most optimal route for this food-grade bacterium. The strategy utilized in this study offers two significant advantages: first, *L. plantarum* plays a key role as the bacterial carrier for protection against environmental degradation and can act as adjuvant that modulates the immune response toward a favorable Th1 response [32–34]. Secondly, the extracellular attachment approach from *E. coli* expression host onto *L. plantarum* cell wall ensures that no implementation of GMO was required.

The immunogenicity of the recombinant Lp ACERL and Lp ACERL/IL-12 was determined based on whether the immune responses were skewing to a Th1 or Th2 mediated response. Most of the *M. tuberculosis* antigens are known to illicit an unfavorable response of mix Th1 and Th2 type-cytokines instead of a dominant Th1 response [20] that is more beneficial in developing protective memory and inducing the required phagocytic response to counter the tuberculosis infection. Ghadimi and co-workers used the probiotic *L. plantarum* to act as an adjuvant for modulating the T-helper cells (Th1, Th2, Th3, and Treg) with their associated cytokine profiles to being skewed to a Th1 mediated response [33]. Meanwhile, Niers and co-workers reported that various specific strains of the lactobacilli and bifidobacteria were able to reduce the production of Th2 type-cytokines (IL-5 and IL-13) [35] while Pochard and others also claimed that the LAB of different strains help to modulate the Th1/Th2 balance by increasing the production of Th1 type-cytokine such as IFN-γ whilst reducing Th2 type-cytokine (IL-4 and IL-5) production from allergic PBMCs specifically re-stimulated with the dust mite allergen [36]. Although the actual mechanisms by which LAB mediate adjuvant activity are yet to be discovered in detail, the effect of LAB in functioning as adjuvant maybe due to its ability to improve antigen presentation and support preferential differentiation of mucosal lymphocytes for the production of protective antibody [37]. This effect by LAB has enhanced the activation of immune responses especially at the mucosal sites such as the NALT and GALT where frequent encounter of antigen occurs. Mohamadzadeh and colleagues demonstrated that the LAB such as *L. plantarum, L. johnsonii,* and *L. gasseri* affected the up-regulation of secretion of Th1 type-cytokines (IL-12, IL-18, IFN-γ) but not Th2 (IL-4, IL-13) [38], suggesting a dominant Th1 polarization. Therefore, a more in depth understanding on the comprehensive mechanisms of action of the LAB is needed to ensure that a more efficient strategy that focuses on vital immune pathways for the development of LAB-type vaccine can be unitized.

**Fig. 6:**
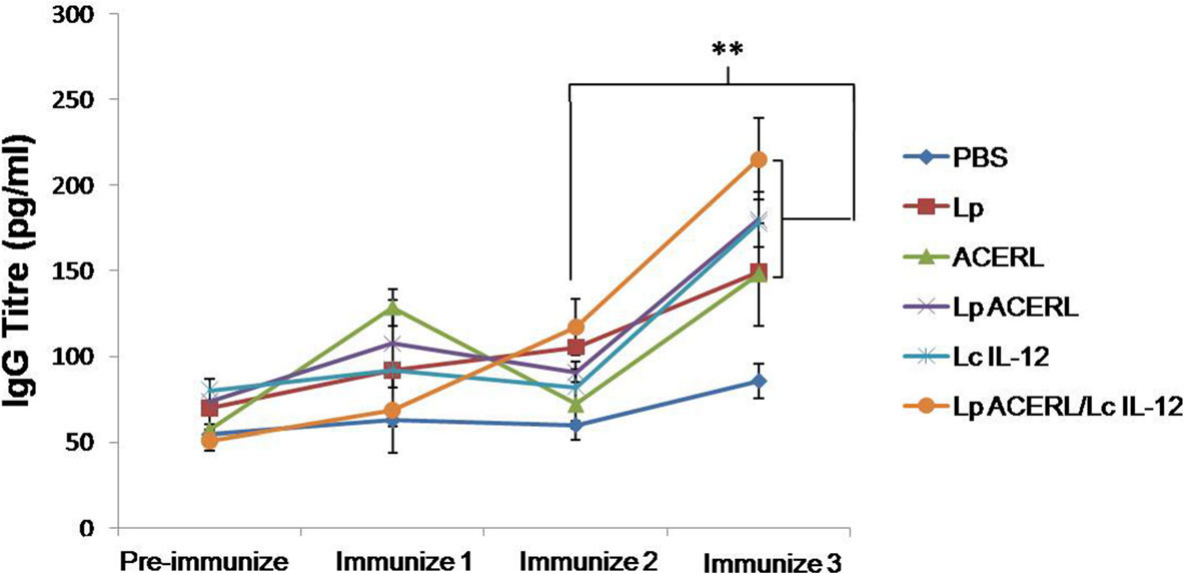
Time-course of the serum total IgG antibody response against Lp ACERL, Lp ACERL/LcIL-12, ACERL, LcIL-12, Lp and PBS immunization. Balb/c mice were immunized thrice via oral administration according to the immunization table of Table 4. Mice were sample bled at 21, 35 and 42 days for determining the total IgG antibodies. Control mice of Balb/c mice immunized with PBS and *L. plantarum* were also sample bled. Each sample was analyzed in triplicates. Results are presented as mean ± SD of the mean (pg/ml) with mean values of *p* < 0.01 = ** between the groups of thrice immunized mice (ACERL, Lp ACERL and Lp ACERL/LcIL-12 group) with the groups of twice immunized mice are significantly different

Investigations on the total serum IgG antibody for the oral group were performed to determine the initial impact on the immune response of Lp ACERL, Lp ACERL/LcIL-12 and its control after several immunizations. It was postulated that a more effective response for Lp ACERL might be achieved by the co-administration with an adjuvant, specifically the bacterial adjuvant *L. lactis* secreting mouse cytokine IL-12 (LcIL-12). The rationale in utilizing LcIL-12 was based on the premise of previous studies that showed potent adjuvant and immunomodulatory effect by LcIL-12 to improve the vaccine outcome [39–41]; IL-12 cytokine is involved in the stimulation of IFN-γ secretion, inhibition of Th2 and stimulation of Th1 immune responses [41] which are key features in enabling long term protective immunity against tuberculosis. The sample bleeds at days 0, 21, 33 and 45 were examined and measured the next day after each immunization session. In general, serum analysis based on Fig. 6, showed that the Lp ACERL/LcIL-12 group achieved higher IgG titre level after the third immunization compared to the Lp ACERL group with 215.2 ± 23.7 pg/ml and 179.7 ± 16.1 pg/ml respectively. This indicates that higher reactivity was achieved for Lp ACERL/LcIL-12 as compared to Lp ACERL. It was observed that the trends for IgG titre level from serum samples for treatment groups (Lp ACERL and Lp ACERL/LcIL-12) had showed significant increase (*P* < 0.01) after the third immunization in comparison to the second immunizations. The trend showed that Lp ACERL and Lp ACERL/IL-12 was able to induce a significant increase of the IgG level (*P* < 0.01) observed after the third immunization. This trend is typical for vaccination due to the adaptation of the immune system with the vaccine where after the third immunization the reactivity reaction is far improved due to its previous recognition based on the first and second immunization [42]. Therefore, several booster immunizations are required to enable sufficient recognition of the vaccine by the host immune system. The test also confirmed that Lp ACERL as being immunogenic and reactive via the oral route.

**Fig. 7:**
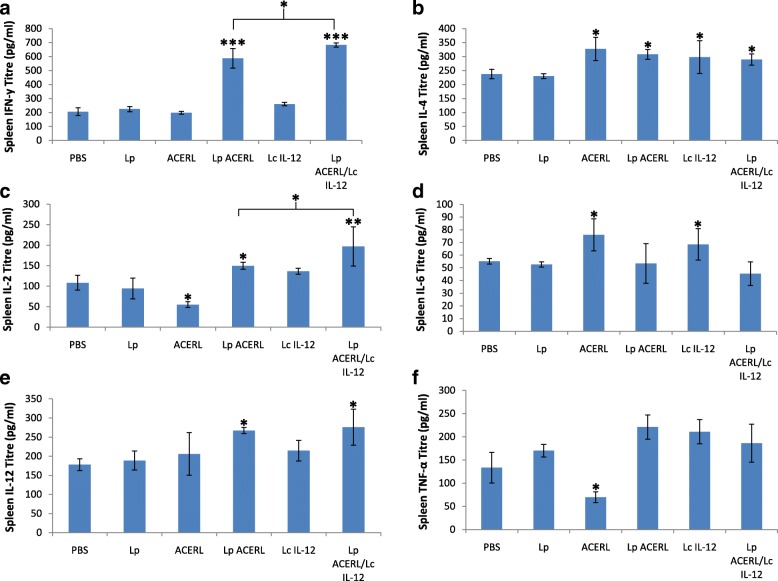
Cytokine response of spleen from orally immunized Balb/c mice. **a** IFN-γ (pg/ml), **b** IL-4 (pg/ml) **c** IL-2 (pg/ml), **d** IL-6 (pg/ml), **e** IL-12 (pg/ml) and **f** TNF-α (pg/ml) detection of spleen from the immunized mice of oral groups were performed using ELISA. Each sample was analyzed in triplicates. Results are presented as mean ± SD of the mean. Mean values of *P* < 0.05 = *, *P* < 0.01 = ** and *P* < 0.001 = *** between the treatment groups and the treatment groups with the PBS and/or Lp control groups were of significant values

**Fig. 8:**
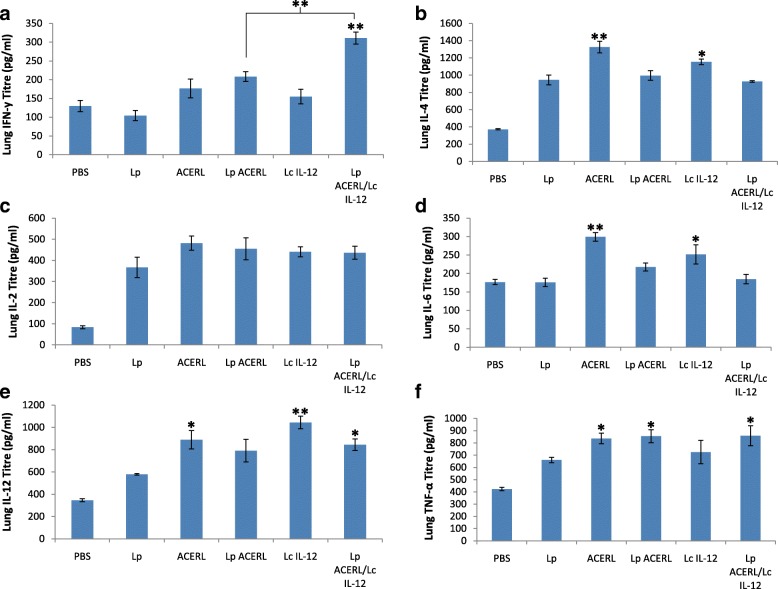
Cytokine response of lung from orally immunized Balb/c mice. **a** IFN-γ (pg/ml), **b** IL-4 (pg/ml) **c** IL-2 (pg/ml), **d** IL-6 (pg/ml), **e** IL-12 (pg/ml) and **f** TNF-α (pg/ml) detection of spleen from the immunized mice of oral groups were performed using ELISA. Each sample was analyzed in triplicates. Results are presented as mean ± SD of the mean. Mean values of *P* < 0.05 = *and *P <* 0.01 = ** between the treatment groups and the treatment groups with the Lp control group were of significant values

**Fig. 9:**
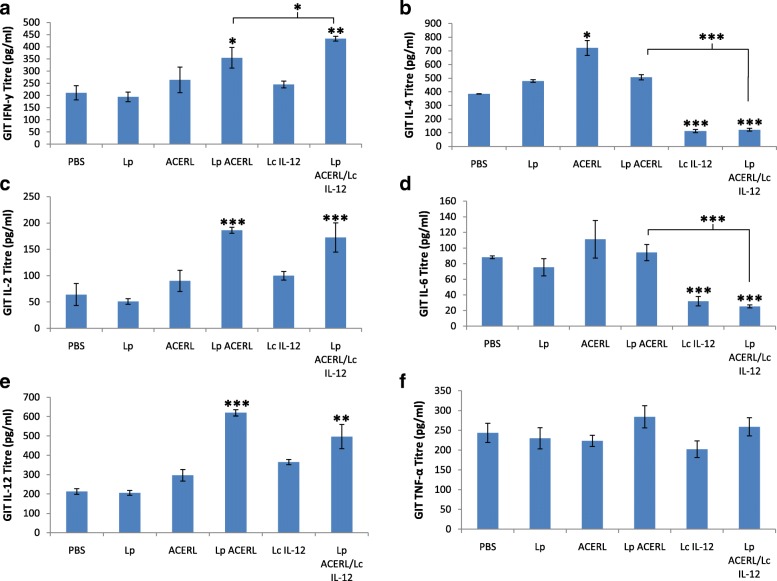
Cytokine response of GIT from orally immunized Balb/c mice. **a** IFN-γ (pg/ml), **b** IL-4 (pg/ml) **c** IL-2 (pg/ml), **d** IL-6 (pg/ml), **e** IL-12 (pg/ml) and **f** TNF-α (pg/ml) detection of spleen from the immunized mice of oral groups were performed using ELISA. Each sample was analyzed in triplicates. Results are presented as mean ± SD of the mean. Mean values of *P* < 0.05 = *, *P* < 0.01 = **and *P* < 0.001 = *** between the treatment groups and the treatment groups with the PBS and/or Lp control groups were of significant values

The analysis of cytokine production in the spleen, lung and GIT was performed in order to establish the effectiveness of Lp ACERL and Lp ACERL/IL-12 immunization via oral route on stimulating the T-cell production in those organ sites which ultimately leads to the activation of memory B cells, the main effector cell responsible for providing the protective memory against tuberculosis. The cytokines that are indicative of Th1 (IFN-y, IL-2 and IL-12) or Th2 (IL-4, IL-6 and TNF-a)-type responses were determined via ELISA from the spleen, lung and GIT samples of the immunized mice. The results from Lp ACERL and Lp ACERL/IL-12 of the orally immunized mice were shown to induce significant increase in IFN-γ (*P* < 0.001) and IL-12(*P* < 0.01) production in spleen (Fig. 7). However, from the lung samples, the changes and increase in IFN-γ production (*P* < 0.01) was observed (Fig. 8) for Lp ACERL/IL-12 but not for Lp ACERL. Although without the increase or changes in IFN-γ, Lp ACERL of oral group only showed an increase in IL-12 and TNF- α without showing strong correlation for either Th1 or Th2 response. Thus, it was postulated that a balance mix of Th1/Th2 responses were induced. Meanwhile, based on the GIT samples (Fig. 9), Lp ACERL and Lp ACERL/IL-12 of orally immunized mice showed significant increase in IFN-γ, IL-2, IL-12 and TNF-α production, a strong indicator of Th1 type-cytokines response. From these findings, Lp ACERL and Lp ACERL/IL-12 immunization via the oral route was shown to illicit strong mucosal and systemic immune responses in spleen, lung and GIT. However, comparison between the cytokine profiling outcome of Lp ACERL with Lp ACERL/LcIL-12 on spleen, lung and GIT samples of orally immunized mice showed Lp ACERL/LcIL-12 improved the tendency for Th1 response as compared to Lp ACERL only. Analysis on the spleen samples (Fig. 7) revealed higher (*P* < 0.05) IFN-γ and IL-2 production in Lp ACERL/LcIL-12 than in Lp ACERL immunized mice group indicating the positive effect of the adjuvant. Lung samples (Fig. 8) showed similar results to Lp ACERL but with one major difference; there was significant increase (*P* < 0.01) in IFN-γ production compared to Lp ACERL and the control groups. This indicates that although different mucosal sites of lung and GIT are spatially compartmentalized, they are immunologically connected, where immune response induction at particular mucosal site is also similarly affected in another distant mucosal tissue [43]. Moreover, the GIT samples (Fig. 9) from Lp ACERL/LcIL-12 showed significant increase (*P* < 0.05) in IFN-γ production while having reduction (*P* < 0.001) inIL-4 and IL-6 production as compared to the Lp ACERL group. It seems that the addition of LcIL-12 had either improved the Th1-type cytokine production or it had reduced the Th2-type cytokines instead. Nonetheless, the effect of LcIL-12 as adjuvant in promoting a bias Th1 response was observed based on the cytokine profiling analysis. Based on these outcomes, it was postulated that the addition of LcIL-12 had improved the Th1-type cytokine production whilst reducing the Th2-type cytokines production.

**Fig. 10:**
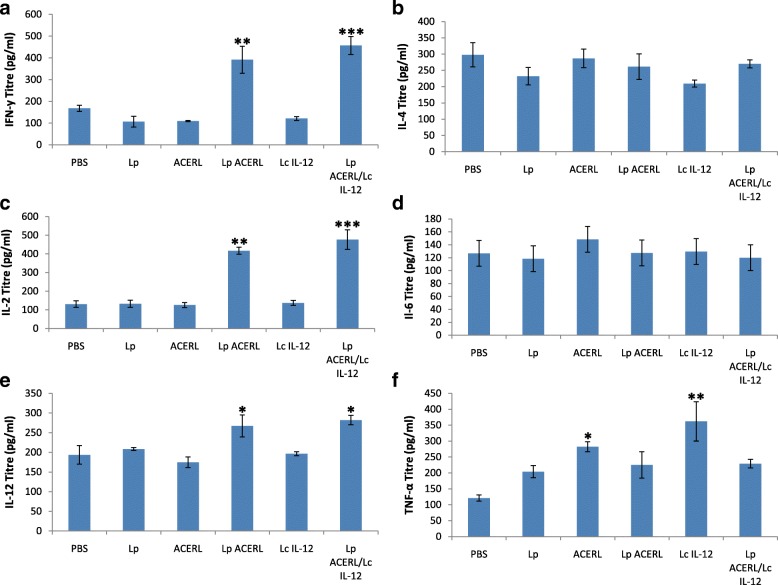
Cytokine response of re-stimulated splenocytes with ACERL antigen after 48 h from orally immunized Balb/c mice groups. **a** IFN-γ (pg/ml), **b** IL-4 (pg/ml) **c** IL-2 (pg/ml), **d** IL-6 (pg/ml), **e** IL-12 (pg/ml) and **f** TNF-α (pg/ml) detection of re-stimulated splenocytes from the immunized mice of the oral groups were measured using ELISA. Each sample was analyzed in triplicates. Results are presented as mean ± SD of the mean. Mean values of *P* < 0.05 = *, *P* < 0.01 = ** and *P* < 0.001 = *** between the immunized mice and the PBS and/or Lp control groups were of significant values

In order to analyze the T-helper type of immune response induced in the different groups of immunized mice, evaluation of the cytokines secreted in cell culture supernatants derived from splenocytes after *in- vitro* re-stimulation with the specific antigen (purified ACERL protein) were performed via ELISA analysis. The secreted levels of the cytokines; IFN-γ, IL-4, IL-2, IL-6, IL-12 and TNF-α as indicated in Fig. 10 showed that significant increase of IFN-γ secretion were detected from orally immunized mice with Lp ACERL (*P* < 0.01) and Lp ACERL/LcIL-12 (*P* < 0.001), in which the latter group possessed the higher IFN-γ level. The IL-2 cytokine level showed similar trend to that of the IFN-γ level production with Lp ACERL (*P* < 0.01) and Lp ACERL/LcIL-12 (*P* < 0.001) having the highest IL-2 level production. Analysis on the cytokine level for IL-4 and IL-6 revealed that no significant changes were observed when compared to its control groups. Levels of IL-12 secreted in splenocyte supernatants increased slightly (*P* < 0.05) for mice group from Lp ACERL and Lp ACERL/IL-12 when compared to the control groups. High levels of TNF-α were also observed in mice immunized with ACERL (*P* < 0.05) and LcIL-12 (*P* < 0.01) groups with the latter group possessing significantly higher TNF-α level. Although the comparison between Lp ACERL/LcIL-12 and Lp ACERL for splenocytes re-stimulation was not statistically significant, Lp ACERL/LcIL-12 showed higher titre values for IFN-γ, IL-2 and IL-12 production as opposed to Lp ACERL of orally immunized mice. Overall, the adjuvant effects of LcIL-12 in improving Lp ACERL to be more reactive and to increase the tendency for a bias Th1 responses were successfully achieved. Furthermore, distal immune response was also observed with the help of LcIL-12 adjuvant.

## Conclusions

The Lp ACERL immunization in mice via the oral administration route was shown to invoke strong Th1-type immune response. The combined results from these studies showed that the candidate vaccine of Lp ACERL had the potential for the rational development and design of effective mucosal tuberculosis vaccine which targets the oral route for vaccine administration. Moreover, comparison between Lp ACERL and Lp ACERL/LcIL-12 of the oral group demonstrated that the positive effect of the LcIL-12 adjuvant is beneficial in improving the Th1 bias response as indicated from the immune response outcome from the cytokine profiling of spleen, lung and GIT that is important for developing protective memory against tuberculosis. The strategy explored here provides a non-invasive mucosal immunization that exploits the benefits from *L. plantarum* that has GRAS status and possessed natural immune-modulating properties. Additional modifications and improvements, such as co-administration with other adjuvants and investigation using various combinations of antigens should be elucidated further to improve this LAB delivery system. The present study provides a proof of concept for a non-GMO tuberculosis vaccine, which may be the strategy in the future for tuberculosis vaccine development.

## Methods

### Bacterial strains and growth conditions

The strains and plasmids used in this study are shown in Table 2. The bacterium *L. plantarum* Pa21 (ATCC 14917) [44] was used as the bacterial delivery vehicle to display the protein of interest on its cell wall. *L. plantarum* Pa21 was propagated without shaking at 37 °C under anaerobic conditions in MRS (Difco, Detroit, MI) broth or on MRS with 1.5% agar. The *E. coli* strain Rossetta (DE3) pLysS was used as the expression host for the intracellular production of candidate vaccine proteins and was propagated at 37 °C in Luria-Bertani, LB (EMD BioSciences, San Diego, CA) or in Terrific broth, TB (EMD BioSciences, San Diego, CA) with shaking at 190 rpm or on LB and TB media supplemented with 1.5% agar. The antibiotic kanamycin (100 μg/ml) was added to broth or agar media used for growing *E. coli* Rossetta harbouring pRSF:Duet-1 expression plasmid. Genetically modified *L. lactis* NZ9000 strains [38] containing mouse IL-12 in the pNZ8048 was obtained from the lab stock and was a generous courtesy from Mr. Jeevanathan Kalyanasundram (this lab). The *L. lactis*/IL-12 uses nisin induction of the PnisA promoter on the pNZ8048 plasmid to enable the expression and secretion of IL-12. The *L. lactis* strains were typically grown at 30 °C as a standing culture in M17 broth or agar [45] supplemented with 0.5% (*w*/*v*) glucose (GM17) and 7.5 μg/ml chloramphenicol whenever necessary. When screening for *L. lactis* transformants, M17 agar supplemented with 0.5% (w/v) glucose, 0.5 M sucrose and 7.5 μg/ml chloramphenicol was used.

**Table 2:** Bacterial strains and plasmids used/or developed in this study

Strain/Plasmid	Characteristics	Source
Strains		
*L. lactis* NZ9000	MG1363 pepN::nisRK, host strain for all lactococcal plasmids	[44]
*E. coli* Rossetta (DE3) pLysS	Expression host for all *E. coli* vectors	Novagen
Plasmids		
pRSF:Duet-1	~ 3.8 kb, Kan^R^, T7 promoter	Invitrogen
pNZ8048	~ 3.3 kb, Cam^R^	[44]
pJet:ACE	~ 3.6 kb, Ap^R^, pJET1.2 vector derivatives containing fusion *M. tuberculosis* antigen of Ag85B, CFP10 and ESAT6 (ACE) with corresponding RE sites for sub-cloning purpose	IDT
pJet:R0R2	~ 3.3 kb, Ap^R^, pJET1.2 vector derivatives containing fusion *M. tuberculosis* antigen of Rv0475 and Rv2031c (R0R2) with corresponding RE sites for sub-cloning purpose	IDT
pNZ:mIL-12	~ 5.0 kb, Cm^R^, pNZ8048 vector derivatives containing mouse IL-12 gene downstream of PnisA	Gift from JK,IBS, UPM
pRSF:LysM	~ 5.6 kb, Kan^R^, pRSFDuet-1 vector derivatives containing LysM anchor motif with corresponding RE sites for sub-cloning purpose downstream of T7 promoter	This study
pRSF:ACERL	~ 5.6 kb, Kan^R^, pRSFDuet-1 vector derivatives containing fusion *M. tuberculosis* antigen gene of Ag85B, CFP10, ESAT6 Rv0475, Rv2031c and LysM (ACERL) with corresponding RE sites for sub-cloning purpose downstream of T7 promoter	This study

### DNA manipulations and plasmid construction

In order to construct pRSF:ACERL, the LysM anchor motif, ACE and R0R2 genes were first amplified through the use of pre-designed primers as indicated in Table 2 whilst the basic outline of the constructed expression vectors is depicted in Fig. 1. The optimum annealing temperature for LysM, ACE and R0R2 genes were determined by the annealing temperature gradient PCR reaction. The respective sets of primers were expected to amplify the LysM (600 bp), ACE (754 bp) and R0R2 (405 bp) genes from genomic DNA of *L. lactis* NZ9000, extracted using the HiYield Plasmid Mini Kit (Yeastern Biotech, Taiwan) according to manufacturer’s protocol with minor modifications, pJET:ACE and pJET:R0R2 plasmids respectively. Initially plasmid backbone was constructed by the cloning of PCR amplified LysM gene into pRSF:Duet-1 via plasmid-insert digestion (*Pst*I/*Not*I) followed by ligation with a 1:4 ratio of plasmid to insert, yielding pRSF:LysM. Subsequently, gel purified PCR products of ACE and R0R2 were digested with *Kpn*I, then purified and ligated together with 1:1 ratio using T4 ligase to produce the ACER fusion gene. This ligation mixture was used as template for PCR amplification of ACER fusion gene using the respective pre-designed primers as in Table 3. The amplified ACER gene was purified and cloned into pRSF:LysM, both insert and plasmid DNAs were initially double digested with *Bam*HI/*Pst*I and purified before they were ligated with T4 ligase at 14 °C overnight with a 1:4 ratio of plasmid to insert. The ligated product was then transformed into *E. coli* Rossetta pLysS (DE3) host by using heat shock method. Following overnight incubation, several putative positive transformants were selected by resistance to kanamycin and were rapidly screened via colony PCR and was further confirmed with its plasmid subjected to double digestion using *Bam*HI and *Not*I restriction enzymes, after which the PCR-amplified fragment were verified by sequencing. Plasmid pRSF:LysM and pRSF:ACERL were purified using a Wizard™ SV Gel and PCR Clean Up System (Promega, USA) according to the manufacturer’s protocol.

**Table 3:** Primers used for amplification of the ACER and LysM genes

Genes	Sequence 5′ ➔ 3’^a^	Optimal Tm (°C)
ACER	Fwd: **GGATCCG**CTGACCAGCGAGCTGCCGCAA	54
	Rev: **CTGCAG**TGGCTTCCCTTCCGAAACCGC	
ACE	Fwd:**GGATCCG**CTGACCAGCGAGCTGCCGCAA	56
	Rev:**CTGCAG**GATATCAGATCTCGCGTTGTTCAGCTCGGTAGC	
R0R2	Fwd:**GGATCCG**AACCTGCGTGAGCGTGCGGAG	54
	Rev: **CTGCAG**TGGCTTCCCTTCCGAAACCGC	
LysM	Fwd: **CTGCAGGGTACC**ACTACTTATACCGTCAAATC	54
	Rev: **GCGGCCGC**TTATTTTATTCGTAGATACTG	

^a^Restriction enzyme (RE) sites are shown as bold and underlined as either *Bam*HI (GGATCCG), *Pst*I (CTGCAG) or *Not*I (GCGGCCGC)

### Protein expression and extraction

Inoculation of *E. coli* Rossetta harbouring pRSF:ACERL or empty pRSF:Duet-1 (as negative control) into fresh TB medium was performed using overnight cultures and grown to an OD _600nm_ of 0.5. The recombinant *E. coli* were expressed for 6 h with 1 mM/mL IPTG at 30 °C with shaking at 200 rpm. The cells were then harvested by centrifugation at 8000×g for 10 min at 4 °C. At this point, if required, the cells can be stored at -80 °C prior to protein extraction. For extraction, the cell pellets containing the protein of interest were prepared for sonication treatment using the Omni Ruptor 4000 (Omni International, USA) set to 40% power and pulsed for 8 min per sample. After that, the sonicated cell suspensions were centrifuged at 12000×g, for 20 min, at 4 °C. Both supernatant and pellet samples were isolated and further verified for the presence of the protein of interest via SDS-PAGE analysis before proceeding with the protein extraction and purification steps. Later, it was determined that all of the expressed proteins of interest were available only in the cell pellet or inclusion bodies (IB). Thus, further steps to extract the protein of interest from IB were taken using the N-lauryl sarcosine (NLS) treatment.

The insoluble fraction containing IBs was re-suspended in 10 ml solubilizing buffer, 40 mM Tris–HCl, pH 8 with NLS concentrations of 0.5%, 1%, 3% or 5% (*w*/*v*). The suspension was agitated at 180 rpm for 24 h at 20 °C, followed by centrifugation at 4400×g for 20 min at 4 °C. Subsequently, the supernatant was then filter sterilized with 0.45 μm membrane filter before the addition of binding buffer (20 mM imidazole, 40 mM Tris-HCl) to achieve final NLS concentration of 0.1% (w/v). The pH of the supernatant was adjusted to pH 7.4 and was loaded onto a Ni^2+^–NTA column (GE Healthcare, USA) which was equilibrated with the binding buffer prior to use. The protein was finally eluted out with elution buffer containing 500 mM imidazole. The concentration of the eluted protein was determined using Bradford assay and its purity analyzed by SDS-PAGE analysis.

Once the IB (cell pellet) treated with NLS, the soluble form of protein of interest was further purified with His-SpinTrap (GE Healthcare, USA) according to the manufacturer’s protocol. However, before the suspension can applied to the His-Spin Trap column, it needed to be filtered again with 0.4 μm filter (Millipore, Molsheim, France) before the addition of imidazole to a final concentration of 20 mM to prevent unspecific binding of the host protein to the column. The columns used were initially equilibrated with 5–10 column volumes of Binding Buffer (20 mM Na_2_HPO_4_, 500 mM NaCl) of 1× PBS containing 20 mM imidazole at pH 7.4. This His-SpinTrap column was used to bind and trap recombinant protein that possessed the His-tag terminal. The column was centrifuged at 100×g for 30 s at 4 °C for each equilibration step. The equilibrated column was loaded with 500–700 μl of the protein mixture at one time and was repeated until all of the protein mixture has been applied through the column. Afterward, a 5 column volumes of Binding Buffer was applied to the column as washing step before a 300 μl Elution Buffer (20 mM Na_2_HPO_4_, 500 mM NaCl) of 1× PBS containing 500 mM imidazole at pH 7.4 was added to the column as final protein elution step. The eluted proteins were then quantified using the Bradford assay and analyzed via and SDS-PAGE.

### Growth study

Cultivation of *L. plantarum*Pa21 surface displayed the fusion antigen of interest was performed on MRS media. The growth curves were produced by plotting the absorbance values (OD_600nm_) versus times (h) for 30 h. Cell stability for each construct was determined based on the doubling time of post-induction absorbance reading. The calculation of the cell doubling time was performed based on the formula of *log*_*10*_*N2-log*_*10*_*N1 = k(t2-t1)/2.303*, where N2 is cell population at later time (t2), N1 is the cell population at initial time (t1) and k is the growth rate. Once the growth rate is determined, the doubling time or generation time (g) is then identified using the formula of *g = ln2/k = 0.693 k* [46].

### Preparation of the vaccine

A single colony of *L. plantarum* was inoculated into 5 ml MRS broth supplemented with 0.5% glucose (GMRS) followed by overnight incubation at 37 °C. Fresh GMRS (10 ml) was inoculated with the overnight culture (0.1 ml) and grown at 37 °C until the culture reached to OD_600nm_ of 0.5–0.7. The cells (1 x 10^9^cfu/ml) were then harvested at 2000×g for 5 min and the cell pellet was re-suspended in 600 μl of fresh GMRS broth. The bacterial suspension was then mixed with 300 μl of the purified recombinant proteins (0.5 mg/ml) and incubated at 30 °C for 3 h. The cells were precipitated at 2000×g for 5 min and then washed with 1× PBS, 3 times. The binding was analyzed using whole cell ELISA and immunofluorescence staining approaches. Lactobacillus cells mixed with 200 μl of 1× PBS was used as controls.

Candidate vaccines were prepared from *L. plantarum* that have undergo protein fusion antigen binding procedure where the vaccine concentration used throughout this study was 1 × 10^9^ cfu/ml attached with 100 μl 0.5 mg/ml purified antigen of interest. Once prepared, the candidate vaccines were used immediately. Similarly, preparation for a mixture of the candidate vaccine with *L. lactis* secreting mIL-12 was also performed. The recombinant *L. lactis* (LcIL12) was grown in GM17 at 30 °C without shaking. To optimize the nisin promoter stimulation, strains were grown until OD_600nm_ reached 0.6, followed by induction with 10 ng/ml nisin (Sigma-Aldrich) for 6 h. Cellular pellets were then harvested by centrifugation at 4000×g at 4 °C and washed three times with 1× PBS. The pellet was re-suspended in 1× PBS to a final concentration of 1 × 10^9^ CFU before the final step of mixing with 1 × 10^9^ CFU of candidate vaccine.

### Binding stability analysis and verification

To confirm the binding of the expressed recombinant protein of ACERL onto cell wall of *L. plantarum*, whole cell ELISA was utilized. The lactobacilli cells which were formerly incubated with the recombinant proteins of interest were harvested by centrifugation at 2000×g for 10 min and then fixed with 4% (*w*/*v*) paraformaldehyde for 20 min at RT, followed by washing step. The cells were then incubated with blocking solution [3% (w/v) BSA in 1× PBS] for 30 min at RT. Then, the cells were incubated with anti-his monoclonal (Novagen, USA) or anti-ACER specific antibody (Calbiochem, USA) as primary antibody at a ratio of 1:200 in 1% BSA in 1× PBS, followed by 1 h incubation at RT. The cells were then harvested and then incubated with horseradish peroxidase conjugated with anti-chicken antibody as secondary antibody at a ratio of 1:200 in 1% BSA in 1× PBS for 1 h at RT. Following three times washing with 1× PBS, the cells were harvested and finally re-suspended in 200 μl of 1× PBS. Ten μl of the bacterial suspension and 50 μl of substrate (BM Blue, Roche, USA) was mixed in the wells of the ELISA plate and then incubated at RT for 20 min. Finally 50 μl of the stop solution (1 M H_2_SO_4_) was added to end the reaction. The absorbance was measured at OD_450_ nm by using ELISA reader (Multiscan MCC/340 MK II, Lab System). Controls were set using lactobacilli cells incubated with BSA or 1× PBS.

The binding of ACERL to *L. plantarum* was visualized by immunofluorescence microscopy using 20 μl mouse anti-his monoclonal antibody [0.2 μg/μl of His∙Tag® Monoclonal Antibody (Novagen, USA)] as primary antibody and Goat Anti-Mouse Flourescein Conjugated Antibody (Calbiochem, USA) (1 μg/μl) as secondary antibodies prepared by 1:200 dilution in 1% BSA (dissolved in 1× PBS). After the binding procedure with ACERL to *L. plantarum*, the attached lactobacilli cells were harvested by centrifugation at 2000 x g for 10 min; washed with 1× PBS and then re-suspended in 200 μl of 1× PBS. About 20–30 μl of the cells (1 × 10^10^ cfu/ml) was dropped on slides coated with poly-L-lysine; air dried and then washed with 1× PBS. The attached cells were fixed with 4% (*w*/*v*) paraformaldehyde for 20 min at RT, followed by 1× PBS washing. In the next step, the cells were incubated with 3% (w/v) BSA in 1× PBS for 30 min at RT, to block non-specific sites. After washing with 1× PBS, the cells were overlaid with primary polyclonal antibody diluted at ratio 1:200 in 1% BSA in 1× PBS followed by incubation at RT for 60 min. The cells were washed with 1× PBS and then incubated with FITC anti-goat conjugated antibody, diluted at 1:200, at RT for 1 h. Following 3 times washing with 1× PBS, the slide was dried and 10 μl of FlourguardAntifade Mounting Solution (Sigma-Aldrich, USA) was added to keep the fluorescence dye from fading. The slide was viewed and analyzed under phase contrast and fluorescence microscope (Nikon ® Eclipse E200 fluorescence microscope) with immersion oil at 100× magnification.

### Immunization protocol

After the completion of the mice acclimatization period, mice were separated into 6 groups of 7 mice as indicated in Table 4. All mice were immunized by oral gavage 3 times at week 1 (days 1, 2, 3), week 4 (days 27, 28, 29) and week 5 (days 40, 41, 42) with 100 μl of 1 × 10^9^ CFU of the vaccine. The dosage of the vaccine was adapted based on Childers and colleagues [47] while the immunization schedule was based on Corthésy and co-workers [48]. Control mice received identical quantities of PBS, *L. plantarum* or *L. lactis*/mIL-12 like the treatment groups. Blood sample from each group was taken by submandibular bleeding during the immunization period at day 0, 21, 33 and 45. All of the mice were euthanized 13 days after the last immunization. The control groups for oral group were divided into two sets which were referred as the baseline set of Group 1 (PBS) and the reference set of Group 2 (*L. plantarum)* and Group 5 (*L. lactis*/mIL-12). Meanwhile the treatment groups were Group 3 (Lp ACERL), Group 4 (LP ACERL + Lc mIL-12), and Group 6 (ACERL).

**Table 4:** Immunization strategy via oral administration in mice

Group	Mouse Strain	Immunized with:	Dose	Route	No of mice
1	Balb/c	PBS	100 μl	Oral	7
2	Balb/c	*L. plantarum* (Lp)	100 μl of 1 × 10^9^ cfu/ml	Oral	7
3	Balb/c	Lp ACERL	50 μg antigen + 100 μl of 1 × 10^9^ cfu/ml	Oral	7
4	Balb/c	Lp ACERL + mIL-12	50 μg antigen + 50 μl of 1 × 10^9^ cfu/ml + 50 μl of 1 × 10^9^ cfu/ml	Oral	7
5	Balb/c	mIL-12	100 μl of 1 × 10^9^ cfu/ml	Oral	7
6	Balb/c	ACERL	50 μg antigen	Oral	7

### Determination of specific antibody IgG

The titre level of IgG total from blood sera of day 0, 21, 33 and 45 was determined using eBioscience Mouse IgG total ready-SET-Go (eBioscience, Austria). Briefly, Corning Costar 9018 ELISA plate was coated with 100 μl/well of antigen ACERL in coating buffer, seal and incubate overnight at 4 °C. The wells were aspirated and washed twice with 250 μl/well wash buffer (1× PBS, 0.05% Tween-20) and the wells blocked with 200 μl of blocking buffer at room temperature for 2 h. The wells were aspirated and washed three times with wash buffer and 100 μl reconstituted standard and sample in assay buffer was added in triplicate into well and incubated for 2 h at room temperature. The wells were aspirated and washed three times with wash buffer and 100 μl/well of diluted detection antibody was added to all wells for 1 h at RT. Then, the wells were aspirated again and washed three times with wash buffer and 100 μl/well of substrate solution was added to each well and incubated at RT for approximately 15 min and before 100 μl of Stop Solution was added to each well and the plate read at 450 nm. The plate was read using the Bio-Plex Suspension Array System model Bio-Plex 100 System (BioRad, USA) and analysed using the Bio-Plex Manager 4.0 (BioRad, USA) software.

### Re-stimulation of splenocytes from the immunized mice

Approximately 2 × 10^7^ cells/ml of splenocytes from the immunized mice were stimμlated with 100 μl of 0.5 mg/ml ACERL antigen protein in 24-well plates (Nunc, Denmark) at 37 °C and 5% CO_2_ for 48 h. Complete RPMI medium containing only mouse splenocytes cells were included as negative control. Once the incubation has completed, 10 μl of cell culture medium was used for cell viability assay. The rest of the cell culture supernatants was centrifuged at 1200×g for 20 min at 4 °C before being sterilized through filter passage of a 0.2 μm pore size filter (Millipore, Germany) and stored at − 80 °C until needed for cytokine analysis. All experiments were performed in triplicate.

### Cytokine assessment

Samples from the supernatant of the induced splenocytes, sera, spleen, feaces, small intestine and lungs were collected and pooled from each group were quantified for cytokine production of IL-2, IL-4, IL-6, IL-12, TNF-α, and IFN-γ via Sandwich ELISA (BD OptEIA™ set mouse IL-2, IL-4, IL-6, IL-12, TNF-α, and IFN-γ, Germany) method. The detection limit of the assay was 6.25 pg/ml for IL-2, 0.5 pg/ml for IL-4, 5.25 pg/ml for IL-6, 6.25 pg/ml for IL-12, 1 pg/ml for IFN-γ, and 20 pg/ml for TNF- α. Optical density values of the samples were read at 450 and 570 nm on an ELISA plate reader (Molecular Devices, Munich, Germany). Experiments were run in triplicate and repeated at least twice, according to the manufacturer’s protocol. Briefly, ELISA plates (Costar, UK) were coated with capture antibody of either goat anti-mouse IL-2, IL-4, IL-6, IL-12, TNF-α, or IFN-γ per well in a 0.1 M carbonate buffer, pH 9.6, at 4 °C overnight. Following three washes with PBS–Tween-20 (PBST) buffer (PBS pH 7·4 and 0.05% Tween-20), plates were incubated with blocking buffer [PBS, 0.1%, Tween-20, 1% bovine serum albumin (BSA)] for 2 h at 37°. After three washes, the plates were incubated with the serially diluted sera in a blocking buffer at 37 °C for 2 h. Subsequently using the washing buffer, the plates were washed thrice before biotinylated secondary antibody rabbit anti-mouse was added and incubated at 37° for 1 h. Then, streptavidin–peroxidase conjugate (1:10000 dilution) was added after washing and incubated at 37° for 1 h. Finally, the plates were washed thrice and color developed by the addition of tetra methyl benzidine TMB (Pharmingen, USA) at RT for 30 min and absorbance was read at 450 nm. The plate was read using the Bio-Plex Suspension Array System model Bio-Plex 100 System (BioRad, USA) and analsyed using the Bio-Plex Manager 4.0 (BioRad, USA) software. The production and ratio between cytokines of IL-2, IL-4, IL-6, IL-12, TNF-α and IFN-γ was calculated based on the maximum production of each cytokine observed in culture via ELISA. The samples that produce the most significant detection of cytokines (*P* < 0.05, *P* < 0.01 or *P* < 0.001) as compared to the control of Lp or/and PBS groups were investigated.

### Statistical analysis

All animal experiments were independently replicated at least thrice. Statistical significance between two groups was evaluated via the student T-test function of the StatView program, version 5.0 (SAS Institute Inc.; Cary, USA) and the graphs were prepared using GraphPad Prism version 7.00 (La Jolla; California, USA). The results were expressed as the mean ± standard deviation (S.D.) of the mean of combined data from the replicate experiments. To measure the differences among groups, the results were tested for significance by a non-parametric way. Results having *P*-values of less than 0.05 were considered statistically significant.

## Additional files

Additional file 1: **Table S1.** Data set of *L. plantarum* Pa21 carrying ACERL antigen growth profiles. (XLSX 25 kb)

Additional file 2: **Table S2.** Data set of whole cell ELISA of *L. plantarum* attached with ACERL for a four-day period. (XLSX 13 kb)

Additional file 3: **Table S3.** Data set from ELISA for time-course of the serum total IgG antibody response against Lp ACERL, Lp ACERL/LcIL-12, ACERL, LcIL-12, Lp and PBS immunization. (XLSX 16 kb)

Additional file 4: **Table S4.** Data set from cytokine response profiling of spleen for IFN-γ (pg/ml), IL-4 (pg/ml), IL-2 (pg/ml), IL-6 (pg/ml), IL-12 (pg/ml) and TNF-α (pg/ml). (XLSX 47 kb)

Additional file 5: **Table S5.** Data set from cytokine response profiling of lung for IFN-γ (pg/ml), IL-4 (pg/ml), IL-2 (pg/ml), IL-6 (pg/ml), IL-12 (pg/ml) and TNF-α (pg/ml). (XLSX 37 kb)

Additional file 6: **Table S6.** Data set from cytokine response profiling of GIT for IFN-γ (pg/ml), IL-4 (pg/ml), IL-2 (pg/ml), IL-6 (pg/ml), IL-12 (pg/ml) and TNF-α (pg/ml). (XLSX 148 kb)

Additional file 7: **Table S7.** Data set from cytokine response profiling of re-stimulated splenocytes with ACERL antigen after 48 h for IFN-γ (pg/ml), IL-4 (pg/ml), IL-2 (pg/ml), IL-6 (pg/ml), IL-12 (pg/ml) and TNF-α (pg/ml). (XLSX 41 kb)

## Abbreviations

ACERL: Ag85B-CFP10-ESAT-6-Rv0475-Rv203
BCG: Bacilli Calmette–Guérin
GIT: Gastrointestinal tract
GMO: Genetically modified organism
IB: Inclusion body
IFN-γ: Interferon gamma
IL-12: Interleukin 12
IL-2: Interleukin 2
IL-4: Interleukin 4
IL-6: Interleukin 6
LAB: Lactic acid bacteria
LcIL-12: *L. lactis* secreting IL-12
Lp ACERL: *L. plantarum* ACERL
LysM: Lysin motif
NLS: N-lauryl sarcosine
Th1: Type 1 T helper
Th2: Type 2 T helper
TNF-α: Tumor necrosis factor alpha
